# 

**Published:** 1857-11

**Authors:** 


### RECEIPTS ON SUBSCRIPTIONS,

UP TO NOV. 20TH, 1857.

### ILLINOIS.

Dr. E. A. Rogers, ........ VOL. 6, ...... $2 00
" G. A. Robinson, ........ " ...... 2 00
" A. P. Finch, ........ " ...... 2 00
" R. F. Henry, ...... VOLS. 5 & 6, ...... 4 00
" S. N. Noble, ........ VOL. 5, ...... 2 00
" H. A. Raney, ........ VOL. 6, ...... 2 00
" F. Ronalds, ........ VOLS. 5 & 6, ...... 5 00
" J. B. Samuel, ........ VOL. 6, ...... 2 00

### INDIANA.

Dr. L. Frazer, ........ VOL. 6, ...... $2 00

" G. B. Buchtell, ........ " ...... 2 00

" S. B. Kyler, ........ VOLS. 5 & 6, ...... 4 00

" D. Rea, ........ VOL. 6, ...... 2 00

" G. Wooden, ........ " ...... 2 00

" D S. Caylor, ........ " ...... 2 00

" R. A. Cameron, ........ " ...... 2 00

" F. D. Gray, ........ " ...... 2 00

" J. B. Collings, ........ " ...... 2 00

### CONNECTICUT.

Dr. R. H. Brown, ........ VOL. 6, ...... $2 00

### WISCONSIN.

Dr. Wm. W. Miller, ........ VOL. 6, ...... $2 00
" G. S. Barrows, ... VOLS. 4, 5 & 6, ...... 6 00
" S. C. Johnson, ........ VOL. 6, ...... 2 00
" J. H. Leitch, ........ VOL. 7, ...... 2 00
" A. P. Adams, ........ " ...... 2 00
" J. B. Daverman, ... VOLS. 5 & 6, ...... 4 00
" E G. Dyer, ........ VOL. 6, ...... 2 00

### IOWA.

Dr. H. Carpenter, ........ VOL 6, ...... $2 00
" H. Fiske, ........ " ...... 2 00
" J. H. Rauch, ........ VOL. 7, ...... 2 00

### MICHIGAN.

Dr. J. B. W. Lewis, ... VOLS. 5 & 6, ...... $4 00
" W. M. Avery, ........ VOL. 6, ...... 2 00
" J. S. Skinner, ........ " ...... 2 00
" J. Tripp, ........ " ...... 2 00

### CALIFORNIA.

Dr. J. R. Brudway, ........ VOL. 6, ...... $2 00
" C. A. Heathwell, ... VOLS. 5 & 6, ...... 4 00

